# Supplementary material for: Discovery and application of insertion-deletion (INDEL) polymorphisms for QTL mapping of early life-history traits in Atlantic salmon
Source: BMC Genomics. 2010 Mar 8;11:156. doi: 10.1186/1471-2164-11-156 (PMC2838853; doi:10.1186/1471-2164-11-156)
Supplement: Additional file 2 — Information on developed 76 locus single-run INDEL panel in Atlantic salmon. Information on fluorescence labeling, primer concentrations, PCR pooling and links to alignments, INDEL motifs and GENESCAN (Burge and Karlin 1997) predictions of genes/exons are available in html format. [file 1471-2164-11-156-S2.ZIP › Additionalfile2/snpsummary20475.html]

```
Cluster 10995 Contig 1

prev  Summary    Contig List  next
```

Size of Consensus sequence = 1356

Number of sequences = 4

Minimum redundancy = 1

Key

A gi|117433099|gb|EG765323.1|EG765323 EST\_ssal\_sjb\_4737 ssalsjb mixed\_tissue Salmo salar cDNA Salmo salar cDNA clone ssal\_sjb\_011\_356\_fwd 3', mRNA sequence  
B gi|117428890|gb|EG761114.1|EG761114 EST\_ssal\_sjb\_9408 ssalsjb mixed\_tissue Salmo salar cDNA Salmo salar cDNA clone ssal\_sjb\_018\_370\_fwd 3', mRNA sequence  
C gi|117433110|gb|EG765334.1|EG765334 EST\_ssal\_sjb\_4738 ssalsjb mixed\_tissue Salmo salar cDNA Salmo salar cDNA clone ssal\_sjb\_011\_356\_rev 5', mRNA sequence  
D gi|24352554|gb|CA046384.1|CA046384 ssalbrh007011 head Salmo salar cDNA, mRNA sequence

6 SNPs detected

A B C D  cosegregation weighted

432 A G . .   6/6 50.00
435 T A . .   6/6 50.00
642 C - C .   6/6 75.00
643 T - T .   6/6 75.00
644 T - T .   6/6 75.00
1228 . . G A   6/6 50.00
